# Supplementary material for: The effects of genital myiasis on the diversity of the vaginal microbiota in female Bactrian camels
Source: BMC Vet Res. 2022 Mar 5;18:87. doi: 10.1186/s12917-022-03189-5 (PMC8897907; doi:10.1186/s12917-022-03189-5)
Supplement: Supplementary file 5 — Additional file 5. [file 12917_2022_3189_MOESM5_ESM.zip › MPL201709200_16s_yy/Treat1/css/js_css/jsPDF-master.zip!/jsPDF-master/docs/plugins_cell.js.html]

plugins/cell.js - Documentation


## Home

### Classes

- jsPDF

### Global

- addFont
- addHTML
- addMetadata
- addPage
- autoPrint
- CapJoinStyles
- circle
- ellipse
- getFontList
- lines
- lstext
- output
- rect
- roundedRect
- save
- setDisplayMode
- setDrawColor
- setFillColor
- setFont
- setFontSize
- setFontStyle
- setLineCap
- setLineJoin
- setLineWidth
- setPage
- setProperties
- setTextColor
- text
- triangle

# plugins/cell.js

```
/** ====================================================================
 * jsPDF Cell plugin
 * Copyright (c) 2013 Youssef Beddad, youssef.beddad@gmail.com
 *               2013 Eduardo Menezes de Morais, eduardo.morais@usp.br
 *               2013 Lee Driscoll, https://github.com/lsdriscoll
 *               2014 Juan Pablo Gaviria, https://github.com/juanpgaviria
 *               2014 James Hall, james@parall.ax
 *               2014 Diego Casorran, https://github.com/diegocr
 *
 * Permission is hereby granted, free of charge, to any person obtaining
 * a copy of this software and associated documentation files (the
 * "Software"), to deal in the Software without restriction, including
 * without limitation the rights to use, copy, modify, merge, publish,
 * distribute, sublicense, and/or sell copies of the Software, and to
 * permit persons to whom the Software is furnished to do so, subject to
 * the following conditions:
 *
 * The above copyright notice and this permission notice shall be
 * included in all copies or substantial portions of the Software.
 *
 * THE SOFTWARE IS PROVIDED "AS IS", WITHOUT WARRANTY OF ANY KIND,
 * EXPRESS OR IMPLIED, INCLUDING BUT NOT LIMITED TO THE WARRANTIES OF
 * MERCHANTABILITY, FITNESS FOR A PARTICULAR PURPOSE AND
 * NONINFRINGEMENT. IN NO EVENT SHALL THE AUTHORS OR COPYRIGHT HOLDERS BE
 * LIABLE FOR ANY CLAIM, DAMAGES OR OTHER LIABILITY, WHETHER IN AN ACTION
 * OF CONTRACT, TORT OR OTHERWISE, ARISING FROM, OUT OF OR IN CONNECTION
 * WITH THE SOFTWARE OR THE USE OR OTHER DEALINGS IN THE SOFTWARE.
 * ====================================================================
 */

(function (jsPDFAPI) {
    'use strict';
    /*jslint browser:true */
    /*global document: false, jsPDF */

    var fontName,
        fontSize,
        fontStyle,
        padding = 3,
        margin = 13,
        headerFunction,
        lastCellPos = { x: undefined, y: undefined, w: undefined, h: undefined, ln: undefined },
        pages = 1,
        setLastCellPosition = function (x, y, w, h, ln) {
            lastCellPos = { 'x': x, 'y': y, 'w': w, 'h': h, 'ln': ln };
        },
        getLastCellPosition = function () {
            return lastCellPos;
        },
        NO_MARGINS = {left:0, top:0, bottom: 0};

    jsPDFAPI.setHeaderFunction = function (func) {
        headerFunction = func;
    };

    jsPDFAPI.getTextDimensions = function (txt) {
        fontName = this.internal.getFont().fontName;
        fontSize = this.table_font_size || this.internal.getFontSize();
        fontStyle = this.internal.getFont().fontStyle;
        // 1 pixel = 0.264583 mm and 1 mm = 72/25.4 point
        var px2pt = 0.264583 * 72 / 25.4,
            dimensions,
            text;

        text = document.createElement('font');
        text.id = "jsPDFCell";

        try {
            text.style.fontStyle = fontStyle;
        } catch(e) {
            text.style.fontWeight = fontStyle;
        }

        text.style.fontName = fontName;
        text.style.fontSize = fontSize + 'pt';
        try {
            text.textContent = txt;            
        } catch(e) {
            text.innerText = txt;
        }

        document.body.appendChild(text);

        dimensions = { w: (text.offsetWidth + 1) * px2pt, h: (text.offsetHeight + 1) * px2pt};

        document.body.removeChild(text);

        return dimensions;
    };

    jsPDFAPI.cellAddPage = function () {
        var margins = this.margins || NO_MARGINS;

        this.addPage();

        setLastCellPosition(margins.left, margins.top, undefined, undefined);
        //setLastCellPosition(undefined, undefined, undefined, undefined, undefined);
        pages += 1;
    };

    jsPDFAPI.cellInitialize = function () {
        lastCellPos = { x: undefined, y: undefined, w: undefined, h: undefined, ln: undefined };
        pages = 1;
    };

    jsPDFAPI.cell = function (x, y, w, h, txt, ln, align) {
        var curCell = getLastCellPosition();
        var pgAdded = false;

        // If this is not the first cell, we must change its position
        if (curCell.ln !== undefined) {
            if (curCell.ln === ln) {
                //Same line
                x = curCell.x + curCell.w;
                y = curCell.y;
            } else {
                //New line
                var margins = this.margins || NO_MARGINS;
                if ((curCell.y + curCell.h + h + margin) >= this.internal.pageSize.height - margins.bottom) {
                    this.cellAddPage();
                    pgAdded = true;
                    if (this.printHeaders && this.tableHeaderRow) {
                        this.printHeaderRow(ln, true);
                    }
                }
                //We ignore the passed y: the lines may have different heights
                y = (getLastCellPosition().y + getLastCellPosition().h);
                if (pgAdded) y = margin + 10;
            }
        }

        if (txt[0] !== undefined) {
            if (this.printingHeaderRow) {
                this.rect(x, y, w, h, 'FD');
            } else {
                this.rect(x, y, w, h);
            }
            if (align === 'right') {
                if (!(txt instanceof Array)) {
                    txt = [txt];
                }
                for (var i = 0; i < txt.length; i++) {
                    var currentLine = txt[i];
                    var textSize = this.getStringUnitWidth(currentLine) * this.internal.getFontSize();
                    this.text(currentLine, x + w - textSize - padding, y + this.internal.getLineHeight()*(i+1));
                }
            } else {
                this.text(txt, x + padding, y + this.internal.getLineHeight());
            }
        }
        setLastCellPosition(x, y, w, h, ln);
        return this;
    };

    /**
     * Return the maximum value from an array
     * @param array
     * @param comparisonFn
     * @returns {*}
     */
    jsPDFAPI.arrayMax = function (array, comparisonFn) {
        var max = array[0],
            i,
            ln,
            item;

        for (i = 0, ln = array.length; i < ln; i += 1) {
            item = array[i];

            if (comparisonFn) {
                if (comparisonFn(max, item) === -1) {
                    max = item;
                }
            } else {
                if (item > max) {
                    max = item;
                }
            }
        }

        return max;
    };

    /**
     * Create a table from a set of data.
     * @param {Integer} [x] : left-position for top-left corner of table
     * @param {Integer} [y] top-position for top-left corner of table
     * @param {Object[]} [data] As array of objects containing key-value pairs corresponding to a row of data.
     * @param {String[]} [headers] Omit or null to auto-generate headers at a performance cost

     * @param {Object} [config.printHeaders] True to print column headers at the top of every page
     * @param {Object} [config.autoSize] True to dynamically set the column widths to match the widest cell value
     * @param {Object} [config.margins] margin values for left, top, bottom, and width
     * @param {Object} [config.fontSize] Integer fontSize to use (optional)
     */

    jsPDFAPI.table = function (x,y, data, headers, config) {
        if (!data) {
            throw 'No data for PDF table';
        }

        var headerNames = [],
            headerPrompts = [],
            header,
            i,
            ln,
            cln,
            columnMatrix = {},
            columnWidths = {},
            columnData,
            column,
            columnMinWidths = [],
            j,
            tableHeaderConfigs = [],
            model,
            jln,
            func,

        //set up defaults. If a value is provided in config, defaults will be overwritten:
           autoSize        = false,
           printHeaders    = true,
           fontSize        = 12,
           margins         = NO_MARGINS;

           margins.width = this.internal.pageSize.width;

        if (config) {
        //override config defaults if the user has specified non-default behavior:
            if(config.autoSize === true) {
                autoSize = true;
            }
            if(config.printHeaders === false) {
                printHeaders = false;
            }
            if(config.fontSize){
                fontSize = config.fontSize;
            }
            if (config.css && typeof(config.css['font-size']) !== "undefined") {
                fontSize = config.css['font-size'] * 16;
            }
            if(config.margins){
                margins = config.margins;
            }
        }

        /**
         * @property {Number} lnMod
         * Keep track of the current line number modifier used when creating cells
         */
        this.lnMod = 0;
        lastCellPos = { x: undefined, y: undefined, w: undefined, h: undefined, ln: undefined },
        pages = 1;

        this.printHeaders = printHeaders;
        this.margins = margins;
        this.setFontSize(fontSize);
        this.table_font_size = fontSize;

        // Set header values
        if (headers === undefined || (headers === null)) {
            // No headers defined so we derive from data
            headerNames = Object.keys(data[0]);

        } else if (headers[0] && (typeof headers[0] !== 'string')) {
            var px2pt = 0.264583 * 72 / 25.4;

            // Split header configs into names and prompts
            for (i = 0, ln = headers.length; i < ln; i += 1) {
                header = headers[i];
                headerNames.push(header.name);
                headerPrompts.push(header.prompt);
                columnWidths[header.name] = header.width *px2pt;
            }

        } else {
            headerNames = headers;
        }

        if (autoSize) {
            // Create a matrix of columns e.g., {column_title: [row1_Record, row2_Record]}
            func = function (rec) {
                return rec[header];
            };

            for (i = 0, ln = headerNames.length; i < ln; i += 1) {
                header = headerNames[i];

                columnMatrix[header] = data.map(
                    func
                );

                // get header width
                columnMinWidths.push(this.getTextDimensions(headerPrompts[i] || header).w);
                column = columnMatrix[header];

                // get cell widths
                for (j = 0, cln = column.length; j < cln; j += 1) {
                    columnData = column[j];
                    columnMinWidths.push(this.getTextDimensions(columnData).w);
                }

                // get final column width
                columnWidths[header] = jsPDFAPI.arrayMax(columnMinWidths);
                
                //have to reset
                columnMinWidths = [];
            }
        }

        // -- Construct the table

        if (printHeaders) {
            var lineHeight = this.calculateLineHeight(headerNames, columnWidths, headerPrompts.length?headerPrompts:headerNames);

            // Construct the header row
            for (i = 0, ln = headerNames.length; i < ln; i += 1) {
                header = headerNames[i];
                tableHeaderConfigs.push([x, y, columnWidths[header], lineHeight, String(headerPrompts.length ? headerPrompts[i] : header)]);
            }

            // Store the table header config
            this.setTableHeaderRow(tableHeaderConfigs);

            // Print the header for the start of the table
            this.printHeaderRow(1, false);
        }

        // Construct the data rows
        for (i = 0, ln = data.length; i < ln; i += 1) {
            var lineHeight;
            model = data[i];
            lineHeight = this.calculateLineHeight(headerNames, columnWidths, model);

            for (j = 0, jln = headerNames.length; j < jln; j += 1) {
                header = headerNames[j];
                this.cell(x, y, columnWidths[header], lineHeight, model[header], i + 2, header.align);
            }
        }
        this.lastCellPos = lastCellPos;
        this.table_x = x;
        this.table_y = y;
        return this;
    };
    /**
     * Calculate the height for containing the highest column
     * @param {String[]} headerNames is the header, used as keys to the data
     * @param {Integer[]} columnWidths is size of each column
     * @param {Object[]} model is the line of data we want to calculate the height of
     */
    jsPDFAPI.calculateLineHeight = function (headerNames, columnWidths, model) {
        var header, lineHeight = 0;
        for (var j = 0; j < headerNames.length; j++) {
            header = headerNames[j];
            model[header] = this.splitTextToSize(String(model[header]), columnWidths[header] - padding);
            var h = this.internal.getLineHeight() * model[header].length + padding;
            if (h > lineHeight)
                lineHeight = h;
        }
        return lineHeight;
    };

    /**
     * Store the config for outputting a table header
     * @param {Object[]} config
     * An array of cell configs that would define a header row: Each config matches the config used by jsPDFAPI.cell
     * except the ln parameter is excluded
     */
    jsPDFAPI.setTableHeaderRow = function (config) {
        this.tableHeaderRow = config;
    };

    /**
     * Output the store header row
     * @param lineNumber The line number to output the header at
     */
    jsPDFAPI.printHeaderRow = function (lineNumber, new_page) {
        if (!this.tableHeaderRow) {
            throw 'Property tableHeaderRow does not exist.';
        }

        var tableHeaderCell,
            tmpArray,
            i,
            ln;

        this.printingHeaderRow = true;
        if (headerFunction !== undefined) {
            var position = headerFunction(this, pages);
            setLastCellPosition(position[0], position[1], position[2], position[3], -1);
        }
        this.setFontStyle('bold');
        var tempHeaderConf = [];
        for (i = 0, ln = this.tableHeaderRow.length; i < ln; i += 1) {
            this.setFillColor(200,200,200);

            tableHeaderCell = this.tableHeaderRow[i];
            if (new_page) {
                this.margins.top = margin;
                tableHeaderCell[1] = this.margins && this.margins.top || 0;
                tempHeaderConf.push(tableHeaderCell);
            }
            tmpArray = [].concat(tableHeaderCell);
            this.cell.apply(this, tmpArray.concat(lineNumber));
        }
        if (tempHeaderConf.length > 0){
            this.setTableHeaderRow(tempHeaderConf);
        }
        this.setFontStyle('normal');
        this.printingHeaderRow = false;
    };

})(jsPDF.API);
```

  

Documentation generated by JSDoc 3.4.2 on Sun Oct 09 2016 11:08:27 GMT+0100 (BST) using the docdash theme.
